# Supplementary material for: Environmental Exposure to Persistent Organic Pollutants and Its Association with Endometriosis Risk: Implications in the Epithelial–Mesenchymal Transition Process
Source: Int J Mol Sci. 2024 Apr 17;25(8):4420. doi: 10.3390/ijms25084420 (PMC11050161; doi:10.3390/ijms25084420)
Supplement: Supplementary file 1 [file ijms-25-04420-s001.zip › Supplementary Figure S1.pptx]

## Slide 1
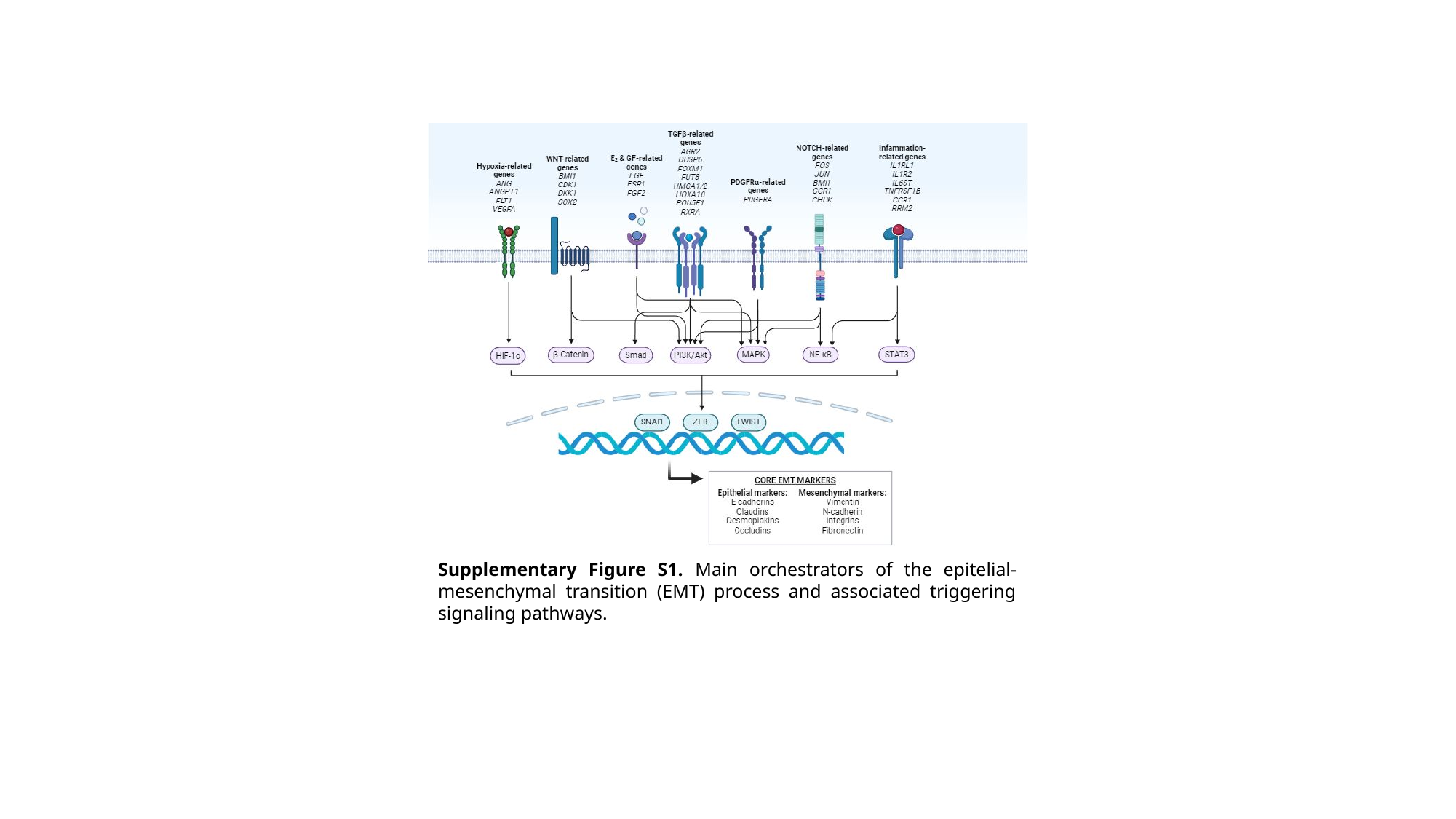

Supplementary Figure S1. Main orchestrators of the epitelial-mesenchymal transition (EMT) process and associated triggering signaling pathways.
